# Geographic variation in body size and its relationship with environmental gradients in the Oriental Garden Lizard, *Calotes versicolor*

**DOI:** 10.1002/ece3.4007

**Published:** 2018-04-02

**Authors:** Xiaomei Wei, Linmiao Yan, Chengjian Zhao, Yueyun Zhang, Yongli Xu, Bo Cai, Ni Jiang, Yong Huang

**Affiliations:** ^1^ Guangxi Botanical Garden of Medicinal Plants Nanning Guangxi China; ^2^ Guangxi Dongli Mechanic School Nanning Guangxi China; ^3^ Department of Herpetology Chengdu Institute of Biology Chinese Academy of Sciences Chengdu Sichuan China; ^4^ Guangxi Key Laboratory of Medicinal Resources Protection and Genetic Improvement Nanning Guangxi China

**Keywords:** Bergmann's rule, climate factors, sexual size dimorphism, temperature seasonality

## Abstract

Patterns of geographic variation in body size are predicted to evolve as adaptations to local environmental gradients. However, many of these clinal patterns in body size, such as Bergmann's rule, are controversial and require further investigation into ectotherms such as reptiles on a regional scale. To examine the environmental variables (temperature, precipitation, topography and primary productivity) that shaped patterns of geographic variation in body size in the reptile *Calotes versicolor*, we sampled 180 adult specimens (91 males and 89 females) at 40 locations across the species range in China. The MANOVA results suggest significant sexual size dimorphism in *C. versicolor* (*F*_23,124_ = 11.32, *p* < .001). Our results showed that *C. versicolor* failed to fit the Bergmann's rule. We found that the most important predictors of variation in body size of *C. versicolor* differed for males and females, but mechanisms related to heat balance and water availability hypotheses were involved in both sexes. Temperature seasonality, precipitation of the driest month, precipitation seasonality, and precipitation of the driest quarter were the most important predictors of variation in body size in males, whereas mean precipitation of the warmest quarter, mean temperature of the wettest quarter, precipitation seasonality, and precipitation of the wettest month were most important for body size variation in females. The discrepancy between patterns of association between the sexes suggested that different selection pressures may be acting in males and females.

## 1 INTRODUCTION

Understanding the evolution of organismal traits such as body size has been a long‐standing goal in evolutionary biology. The geographic variation in body size has been assumed to be a complex consequence of both proximate (developmental) and ultimate (evolutionary) causes, including differences in geography, climate, resource quality and availability, size‐specific predation, population competition, sexual selection, etc. (Berven & Gill, 1983; Cooper & Purvis, 2010; Hileman et al., 2017; Mayr, 1956; Sandland & Minchella, 2004). Patterns of geographic variations in body size have been corroborated for a large number of taxa (Maestri, Luza, Barros, Hartz, & Ferrari, 2016) and are more likely to be exhibited in large geographic ranges (Meiri & Thomas, 2011).

One commonly recognized clinal pattern in body size across a broad geographic range was postulated by Karl Bergmann, now termed Bergmann's rule, which assumes that individuals on land tend to increase in size with increasing latitude and related ecological processes (Bergmann, 1847; Maestri et al., 2016). This rule seems to fit well for endotherm such as land birds and mammals (Ashton, 2002; O'Keefe, Meachen, Fet, & Brannick, 2013); however, the universality of Bergmann's rule is still controversial for ectotherms such as reptiles (Adams & Church, 2008; Ashton & Feldman, 2003; Pincheira‐Donoso, Hodgson, & Tregenza, 2008). Despite the number of studies on Bergmann's rule, most have considered only the relationship between body size variation and only temperature or latitude across broad ranges (Ashton & Feldman, 2003; Atkinson, 1994; Blackburn, Gaston, & Loder, 1999; Boaratti & Da Silva, 2016). Through various empirical and theoretical developments, relationships between body size and environmental factors such as precipitation, topography, resource quality, and availability within species at the regional level require further investigation because of local adaptation and/or phenotypic plasticity (Ashton & Feldman, 2003; Boaratti & Da Silva, 2016; Muñoz, Wegener, & Algar, 2014; Phillimore, Hadfield, Jones, & Smithers, 2010). These regional‐scale studies provided insight into an organisms' response to environmental changes (Michael et al., 2014).

Geographic variation in lizard body size is assumed to be shaped by different environmental gradients and ultimately reflect adaptation to local environmental conditions (Iglesias, Tracy, Bedford, & Christian, 2012). For example, association between food quality and primary productivity could impose a selective pressure on body size, where a decrease in food quality usually leads to a smaller size because of slow growth and vice versa (Berrigan & Charnov, 1994). In addition, lizards with a small body size are expected to gain and lose heat more quickly than larger animals, thus factors related to thermal regimes such as daily activity patterns could have an impact (Meiri, 2008). In general, it is still difficult to assume a most general mechanism underlying body size–environment relationships of lizards from studies conducted in tropical regions in a regional scale (Boaratti & Da Silva, 2016; Vinarski, 2014).

The Oriental Garden Lizard, *Calotes versicolor*, is a good model organism for studying body size variations and the relationship with environmental gradients because of its tropical–subtropical distribution (from Oman, across southern and Southeast Asia to Indo‐China, the Maldives, Réunion, Mauritius, and the Seychelles) and highly heterogeneous habitats across an elevation range from 80 to 2,000 m (Boulenger, 1912; Günther, 1864; Radder, 2006; Smith, 1935; Zhao, Zhao, & Zhou, 1999). Some studies have shown that *C. versicolor* has a complex evolutionary history and revealed several distinct mitochondrial lineages (Huang et al., 2013; Zug, Brown, Schulte, & Vindum, 2006). Geographic variation in body size has been reported in this species; as in many other animals, sexual differences in size occur in some regions and appear at any life stage (Auffenberg & Rehman, 1993; Ji, Qiu, & Diong, 2002; Prakobkarn, Thirakhupt, & Ngamprasertwong, 2016; Radder, 2006; Radder, Shanbhag, & Saidapur, 2001). However, it is still unclear whether sexual size dimorphism occurs in other parts of its range, including within China, or shows the same patterns (Prakobkarn et al., 2016).

Here, we have investigated body size variation in *C. versicolor* in both males and females. The aims of our study were to: (1) describe sexual dimorphism in size; (2) characterize geographic variation in body size in order to detect local patterns, and (3) determine which factors are likely to have the greatest effect on the evolution of body size within this species, including temperature, cumulative temperature, precipitation, wind, elevation, and normalized difference vegetation index (NDVI). Thus, we investigated four competing hypotheses to explore these relationships according to the study of Boaratti and Da Silva (2016) with minor modifications: (1) the heat balance hypothesis, largely dependent on temperature, argues that larger organisms would be favored in cold environments and vice versa (Olalla‐Tárraga, Rodríguez, & Hawkins, 2007). This could be explained by larger body size enhancing the ability to conserve heat in colder environments due to heat being dissipated more slowly in larger animals as the surface area‐to‐volume ratio decreases (Meiri, 2008); (2) the water availability hypothesis, measured by precipitation, predicts that body size in dry environments would be larger than in moist environments,because a larger animal would have a reduced surface area‐to‐volume ratio, and hence would be less susceptible to desiccation (Ashton, 2002; Claussen, 1967); (3) the productivity hypothesis, related to resource availability such as food quality or quality of prey, proposes that the conditions of low habitat productivity might be expected to cause constraints on body size (Boyce, 1978; Ernest, Brown, & Parmenter, 2003); and (4) the topography hypothesis predicts that body size will decrease with elevation (Morrison & Hero, 2003).

## 2 MATERIALS AND METHODS

### 2.1 Ethical statement

Sample collections were carried out in strict accordance with the “Regulation for the Collection of Genetic Resources (HJ 628–2011),” and all practical efforts were made to ameliorate suffering of study specimens throughout this study. This study was approved by the Animal Ethics Committee of Guangxi Botanical Garden of Medicinal Plants, and animal experiments were carried out in line with the institutional guidelines.

### 2.2 Sampled localities and morphological data

**Figure 1:**
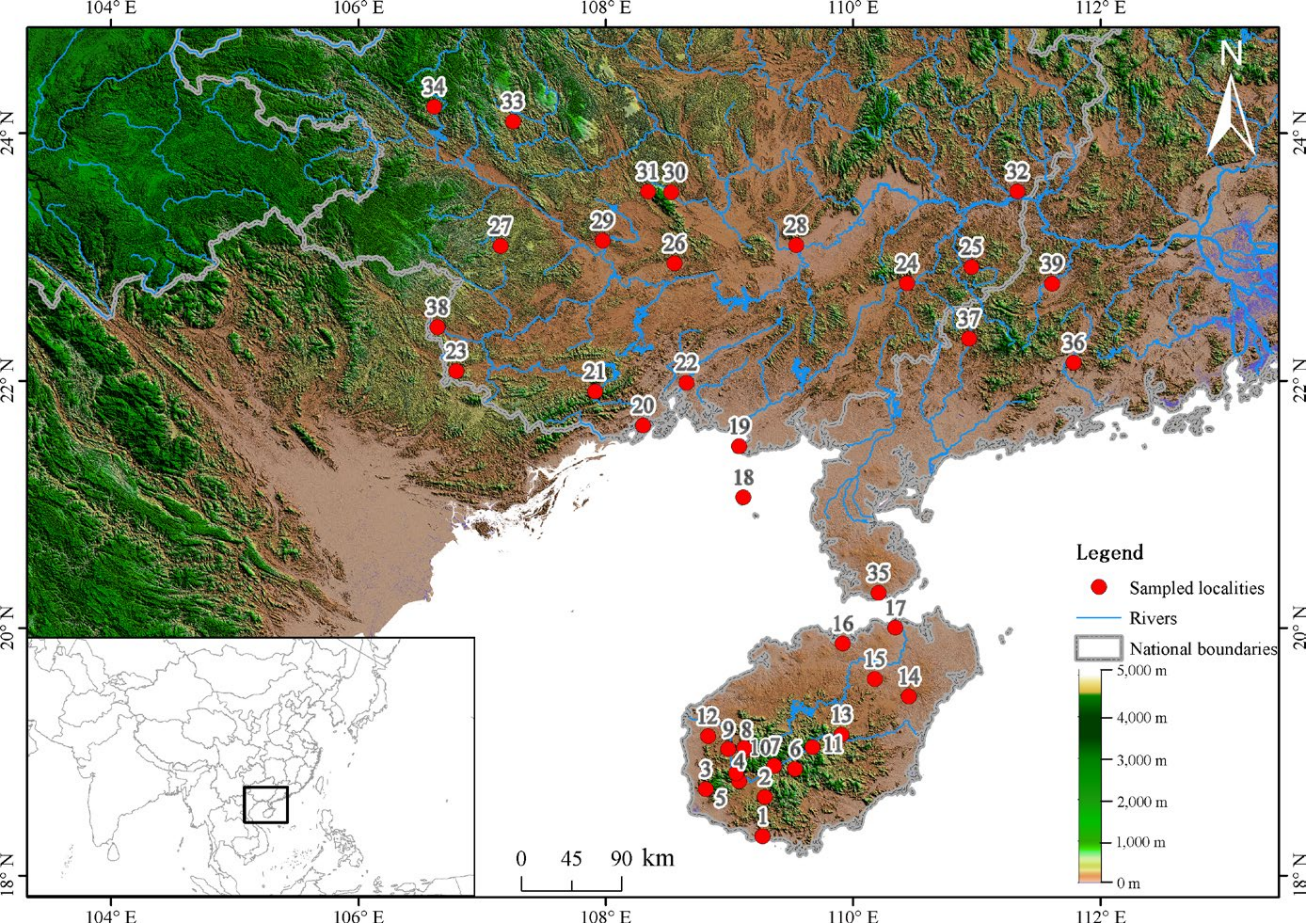
Sampled localities for the *Calotes versicolor*. Samples are numbered following Table 1

Specimens were collected from 40 populations (180 individuals) through several field trips (Figure 1, Table 1). A total of 91 males and 89 females were examined for this study. Those mensural traits possessing terminal endings on bone and along axes with rigorous bony struts reducing compression or bending were taken into account (Zug et al., 2006). The following traits were measured: eye–ear length (EyeEar), eye length (EyeL), head height (HeadH), head length (HeadL), head width (HeadW), interorbital width (Interorb), jaw width (JawW), naris–eye length (NarEye), snout–eye length (SnEye), snout width (SnW), 4th finger length (4FingLng), 4th toe length (4ToeLn), crus length (CrusL), forefoot length (ForefL), hindfoot length (HindfL), lower arm length (LoArmL), snout–vent length (SVL), snout–forelimb length (SnForel), tail height (TailH), tail length (TailL), tail width (TailW), upper arm length (UpArmL), and upper leg length (UpLegL). The abbreviations given in parentheses are those defined by Zug et al. (2006). Each individual's gonads were examined to determine sex and maturity. Maturity in males was based on the presence of sperm (absent in females). Maturity in females was further determined by abdominal dissection (Zug et al., 2006). All traits were reported for the right side and were measured in millimeters using digital vernier calipers.

**Table 1:** Morphological data: Mean ± *SEM* of male (*N* = 91) and female (*N* = 89) adults in 40 populations of *Calotes versicolor*

Number	Populations	Latitude	Longitude	Sex	*N*	SVL
1	Tianya	18.31	109.27	F	4	85.49 ± 1.19
				M	4	90.97 ± 4.13
2	Zhizhong	18.63	109.29	F	3	80.71 ± 0.63
				M	1	—
3	Jianfeng	18.70	108.81	F	2	88.03 ± 0.87
				M	2	77.12 ± 1.50
4	Baoyou	18.76	109.08	F	6	76.31 ± 1.68
				M	2	78.89 ± 0.57
5	Jianbian	18.82	109.06	M	1	—
6	Hongshan	18.86	109.53	F	2	81.93 ± 2.63
				M	3	88.21 ± 3.51
7	Fanyang	18.88	109.36	F	5	86.44 ± 1.33
8	Wangxiaxiang	19.01	109.14	F	8	82.55 ± 1.48
9	Donghe	19.02	108.99	M	4	84.89 ± 0.64
10	Bawangling	19.03	109.12	F	3	84.35 ± 0.42
				M	4	78.94 ± 0.48
11	Hongmao	19.03	109.67	F	4	84.59 ± 2.73
				M	4	82.33 ± 3.71
12	Datian	19.12	108.83	F	5	86.08 ± 0.97
				M	1	—
13	Qiongzhong	19.13	109.91	M	3	75.48 ± 5.35
14	Huangzhu	19.44	19.44	F	1	—
15	Tunchang	19.58	110.18	M	1	—
16	Fushan	19.87	109.92	M	1	—
17	Haikou	20.00	110.34	F	2	85.09 ± 5.09
18	Weizhoudao	21.06	109.11	F	6	90.16 ± 2.94
				M	13	93.11 ± 1.28
19	Yinhai	21.47	109.08	F	2	91.40 ± 0.81
				M	1	—
20	Gangkou	21.64	108.30	F	2	84.09 ± 5.23
				M	1	—
21	Shiwandashan	21.91	107.92	M	1	—
22	Qinnan	21.98	108.65	F	5	90.41 ± 3.67
				M	5	92.79 ± 3.55
23	Buguan	22.07	106.79	M	1	—
24	Rongxi	22.78	110.44	F	2	72.44 ± 1.42
				M	3	92.31 ± 7.52
25	Cenxi	22.91	110.96	F	1	—
				M	3	84.71 ± 6.42
26	Wutang	22.95	108.56	F	2	78.03 ± 5.18
				M	1	—
27	Tiandeng	23.09	107.15	F	3	78.73 ± 5.76
				M	1	—
28	Gangbei	23.09	109.54	F	1	—
				M	4	80.10 ± 0.64
29	Dingdang	23.13	107.98	F	3	82.84 ± 4.03
				M	3	83.79 ± 5.00
30	Yunchun	23.52	108.53	M	3	84.01 ± 6.03
31	Damingshan	23.53	108.34	F	1	—
				M	5	74.56 ± 1.97
32	Wuzhou	23.53	111.33	F	3	80.29 ± 2.33
				M	1	—
33	Naheng	23.95	107.07	M	1	—
34	Bayan	24.09	107.25	F	2	85.00 ± 0.66
				M	3	84.80 ± 5.32
35	Lingyun	24.21	106.61	F	3	95.45 ± 1.47
				M	1	—
36	Haian	20.28	110.21	M	1	—
37	Yangchun	22.14	111.78	F	2	85.68 ± 2.26
				M	3	88.6 ± 1.81
38	Xinyang	22.34	110.94	F	2	87.54 ± 2.49
				M	3	88.89 ± 4.78
39	Shuikou	22.43	106.64	F	1	—
40	Luoding	22.78	111.61	F	3	84.23 ± 0.54
				M	2	80.37 ± 5.77

—, denotes data cannot be calculated due to one sample; SVL, snout–vent length.

### 2.3 Hypotheses and environmental variables

We initially selected 24 environmental variables summarizing temperature, precipitation, elevation, NDVI, wind, and cumulative temperature. We considered the hypotheses proposed by Boaratti and Da Silva (2016) to test the influence of environmental variables on body size in *C. versicolor*. These hypotheses and their associated variables were as follows:

1. the heat balance hypothesis—We used annual mean temperature (BIO1), mean diurnal range (BIO2), isothermality (BIO3), temperature seasonality (BIO4), maximum temperature of the warmest month (BIO5), minimum temperature of the coldest month (BIO6), temperature annual range (BIO7), mean temperature of the wettest quarter (BIO8), mean temperature of the driest quarter (BIO9), mean temperature of the warmest quarter (BIO10), mean temperature of the coldest quarter (BIO11), mean annual wind speed (WIND), mean annual sum of effective temperature (≥0°C) (AAT0DEM), and mean annual solar radiation (RAD) as the predictor variables. These variables (BIO1 to BIO11) were obtained at a resolution of 30 s from the WorldClim bioclimatic database (http://www.worldclim.org/) (Hijmans, Cameron, Parra, Jones, & Jarvis, 2005). WIND was with 500 m^2^ resolutions available at http://www.geodata.cn/Portal. WIND and AAT0DEM, both at a resolution of 500 m^2^, and RAD at a resolution of 1 km^2^ were available at http://www.geodata.cn/Portal.
2. the water availability hypothesis—We used total annual precipitation (BIO12), precipitation of the wettest month (BIO13), precipitation of the driest month (BIO14), precipitation seasonality (BIO15), precipitation of the wettest quarter (BIO16), precipitation of the driest quarter (BIO17), precipitation of the warmest quarter (BIO18), and precipitation of the coldest quarter (BIO19) as predictor variables. These variables were obtained at a resolution of 30 s from the WorldClim bioclimatic database (Hijmans et al., 2005).
3. the productivity hypothesis—We used mean normalized difference vegetation index (NDVI) as the predictor variable. The mean NDVI represents an estimate of the density or sparseness of vegetation in each grid cell and is therefore a proxy for the biotic competitive environment (Nakazato, Warren, & Moyle, 2010). NDVI was available at https://lpdaac.usgs.gov/get_data and is maintained by the NASA Land Processes Distributed Active Archive Center, USGS/Earth Resources Observation and Science Center, Sioux Falls, South Dakota.
4. the topography hypothesis—We used mean elevation (ELE) as the predictor variables. Elevation at resolution of 30 s was available at http://eros.usgs.gov/.

### 2.4 Statistical analyses

We used multivariate analysis of variance (MANOVA) to determine whether there were significant differences in both the sexes, with sex as the independent variable and the 23 morphological traits as the dependent variables. Morphological variables for each sex were calculated through principal component analyses (PCA) on the correlation matrix. However, because the value loading on first principal component (PC1) was low (56.8% and 46.8% of variance for males and females, respectively, Table S1), this axis does not represent size. Thus, we used male and female adult SVL (sample size equal to or more than 3 for each population) as a proxy in subsequent analyses to examine relationships with candidate environmental variables. SVL is a widely used measure of body size in squamate reptiles and is positively associated with, for example, mass and ecological and life‐cycle traits (Pincheira‐Donoso et al., 2008).

We used simple regression and stepwise multiple regression analyses to determine the relationship between SVL and environmental variables. Due to species dispersal, gene flow, or other variables, samples collected from identical or nearby localities are not expected to be independent (i.e., spatial autocorrelation; locations close to each other exhibit more similar values than those further apart) (Boaratti & Da Silva, 2016; Dormann et al., 2007). We generated alternative models using spatial eigenvector mapping (SEVM) obtained by principal coordinates neighbor matrices to account for spatial autocorrelation (Diniz‐Filho & Bini, 2005). These eigenvectors are then orthogonal variables that capture the geometry of the grid covering the study area on different scales and thus can be incorporated into multiple regression models in different ways (Rangel, Diniz‐Filho, & Bini, 2006). It has been demonstrated that SEVM with small a number of eigenvectors (i.e., small L) greatly reduces model misspecification errors and increases model accuracy (Murakami & Griffith, 2017; Thayn & Simanis, 2013; Tiefelsdorf & Griffith, 2007). Thus, SEVM is a popular spatial model in applied studies (Pace et al., 2013). Indeed, many studies also suggested that SEVM can achieve the aim of accounting for spatial autocorrelation in ecological data (Fergnani, Ruggiero, Ceccarelli, Menu, & Rabinovich, 2013; Gouveia, Hortal, Cassemiro, Rangel, & Diniz‐Filho, 2013; McClain, Allen, Tittensor, & Rex, 2012; Murakami & Griffith, 2017; Yang et al., 2016). A truncation distance of 215.9 km for males and 316.9 km for females, calculated in SAM (Spatial Analysis in Macroecology), version 4.0 (Rangel, Diniz‐Filho, & Bini, 2010), was used to create the spatial filters. These filters were then used as candidate predictor variables together with environmental variables in the full model. Then, to test which models best explain body size variations in *C. versicolor*, we used a sample size‐corrected Akaike information criterion (AICc) to evaluate the goodness of model fit (Burnham & Anderson, 1998). The model with the lowest AICc score was considered the most parsimonious (Burnham & Anderson, 1998). The larger the ΔAICc values, the less likely are the best approximating model in the given model set. In addition, model averaging of estimates using the Akaike weights (wAICc) was used to confront the uncertainty of the model selection (Anderson, Burnham, & Thompson, 2000; Burnham & Anderson, 1998). All analyses were performed separately in SAM (Rangel et al., 2010).

## 3 RESULTS

### 3.1 Patterns of geographic variation of body size

Body size varied widely between adult male and female lizards. MANOVA showed that significant sexual size dimorphism in *C. versicolor* (*F*_23,124_ = 11.32, *p* < .001; Tables 1 and S2); thus, further analyses were performed separately in males and females. We found a significant negative relationship between body size variation and latitude in females (slope ± *SE* = −0.30 ± 0.04, *t* = −6.69, *p* < .001), while this relationship was not significant in males (slope ± *SE* = −0.01 ± 0.06, *t* = −0.23, *p* = .82), indicating an inverse Bergmann cline.

### 3.2 Relationships between body size and candidate variables

Body size variations in both males and females were associated with precipitation and temperature variables. Based on the ΔAICc for the best estimates of the variables, the results showed that body size variation in males, it was significantly negatively related to temperature seasonality (BIO4) (*p* = .02, *r* ^2^ = .34, Table 2), precipitation of the driest month (BIO14) (*p* = .02, *r* ^2^ = .34), and precipitation of the driest quarter (BIO17) (*p* = .04, *r* ^2^ = .33), respectively (Table 2), and positively correlated with precipitation seasonality (BIO15) (*p* = .04, *r* ^2^ = .33, Table 2). In females, the body size variation showed a positive association with mean precipitation of warmest quarter (BIO18) (*p* = .01, *r* ^2^ = .17), mean temperature of the wettest quarter (BIO8) (*p* = .02, *r* ^2^ = .14), precipitation seasonality (BIO15) (*p* = .02, *r* ^2^ = .14), and precipitation of the wettest month (BIO13) (*p* = .03, *r* ^2^ = .13, Table 2). However, when all the significant environmental variables (Table 2) were included to explain male SVL variations in multiple regression analyses using eigenvector‐based spatial filtering, only temperature seasonality remained significant (*r* ^2^ = .38, *p* < .05). Similar analyses for females, only precipitation of warmest quarter remained significant (*r* ^2^ = .33, *p* = .009).

**Table 2:** Spatial eigenvector mapping (SEVM) models predicting the relationship between environmental gradients and body size geographic distribution of *Calotes versicolor*

Predictor variables	AICc	ΔAICc	wAICc	Predictors only *R* ^2^	Predictors + filters *R* ^2^	*b*	*p*‐Value
Males							
BIO4	529.81	0.00	0.18	.01	.34	−0.01	.02
BIO14	530.51	0.70	0.13	.00	.34	−0.71	.03
BIO15	530.90	1.09	0.10	.09	.33	0.71	.04
BIO17	531.12	1.31	0.09	<.001	.33	−0.21	.04
BIO19	531.30	1.49	0.08	.01	.33	−0.12	.05
BIO7	532.43	2.62	0.05	.00	.32	−2.44	.09
BIO9	532.67	2.86	0.04	.00	.32	1.37	.10
BIO11	533.07	3.26	0.03	.00	.32	1.48	.12
BIO3	533.44	3.63	0.03	.04	.31	1.19	.15
BIO6	533.50	3.68	0.03	.01	.31	1.24	.16
NDVI	533.68	3.87	0.03	.11	.31	−20.90	.17
BIO1	534.28	4.47	0.02	.02	.30	1.19	.25
RAD	534.56	4.75	0.02	.05	.30	<0.001	.30
BIO8	534.58	4.77	0.02	.12	.30	1.30	.31
ELE	534.80	4.99	0.01	.14	.30	−0.01	.36
BIO10	535.20	5.38	0.01	.07	.30	0.73	.49
WIND	535.27	5.46	0.01	.16	.29	1.10	.52
AAT0DEM	535.31	5.50	0.01	.03	.29	<0.001	.54
BIO2	535.51	5.70	0.01	.11	.29	−1.11	.65
BIO5	535.53	5.72	0.01	.03	.29	0.46	.67
BIO12	535.57	5.76	0.01	.00	.29	0.00	.70
BIO16	535.71	5.90	0.01	.04	.29	0.00	.88
BIO18	535.73	5.92	0.01	.07	.29	<0.001	.94
BIO13	535.73	5.92	0.01	.09	.29	0.00	.97
Females							
BIO18	427.46	0.00	0.35	.09	.17	0.03	.01
BIO8	429.54	2.08	0.12	.13	.14	2.78	.02
BIO15	429.65	2.19	0.12	.01	.14	0.62	.02
BIO13	430.58	3.12	0.07	.05	.13	0.07	.04
RAD	432.00	4.54	0.04	.00	.11	<0.001	.09
BIO19	432.01	4.55	0.04	.01	.11	−0.10	.09
WIND	432.12	4.66	0.03	.03	.11	1.84	.09
BIO3	432.42	4.96	0.03	.07	.10	−0.61	.11
BIO4	433.28	5.82	0.02	.05	.09	0.00	.18
BIO14	433.59	6.13	0.02	.01	.09	−0.27	.22
BIO17	433.67	6.21	0.02	.01	.08	−0.10	.23
BIO2	433.70	6.24	0.02	.01	.08	−2.99	.23
ELE	434.01	6.55	0.01	.01	.08	−0.01	.29
BIO16	434.15	6.69	0.01	.04	.08	0.01	.32
BIO9	434.22	6.76	0.01	.05	.08	−0.66	.33
NDVI	434.24	6.79	0.01	.06	.08	−13.23	.34
BIO10	434.64	7.18	0.01	.02	.07	0.76	.46
BIO5	434.70	7.24	0.01	.02	.07	0.74	.48
BIO7	435.05	7.59	0.01	.02	.06	0.46	.67
BIO11	435.08	7.62	0.01	.04	.06	−0.32	.69
BIO12	435.14	7.68	0.01	.02	.06	0.00	.74
BIO1	435.19	7.73	0.01	.01	.06	0.25	.81
BIO6	435.20	7.74	0.01	.02	.06	0.18	.82
AAT0DEM	435.24	7.78	0.01	.02	.06	<0.001	.91

Predictor variables: BIO1, annual mean temperature; BIO2, mean diurnal range; BIO3, isothermality; BIO4, temperature seasonality; BIO5, maximum temperature of the warmest month; BIO6, minimum temperature of the coldest month; BIO7, temperature annual range; BIO8, mean temperature of the wettest quarter; BIO9, mean temperature of the driest quarter; BIO10, mean temperature of the warmest quarter; BIO11, mean temperature of the coldest quarter; BIO12, annual precipitation; BIO13, precipitation of the wettest month; BIO14, precipitation of the driest month; BIO15, precipitation seasonality; BIO16, precipitation of the wettest quarter; BIO17, precipitation of the driest quarter; BIO18, precipitation of the warmest quarter; and BIO19, precipitation of the coldest quarter; NDVI, normalized difference vegetation index; ELE, elevation; WIND, mean annual wind speed; AAT0DEM, mean annual sum of effective temperature (≥0°C); RAD, mean annual solar radiation. AICc, Akaike information criterion corrected for small samples; ΔAICc, difference between the interest model and the model with the lowest AICc value; wAICc, AICc weight model that expresses the weight of evidence favoring the model as the best among all the models compared.

## 4 DISCUSSION

### 4.1 Sexual size dimorphism

Sexual differences in body size variation are a widespread phenomenon in lizards, known as sexual size dimorphism (Fairbairn, 1997, 2007; Olsson, Shine, Wapstra, Ujvari, & Madsen, 2002). Our results suggest the presence of sexual size dimorphism in *C. versicolor* adults, in accordance with previous work in this species (Diong, Chou, & Lim, 1994; Ji et al., 2002; Prakobkarn et al., 2016; Qiu, Ma, & Ji, 2001; Radder et al., 2001) and other lizards (e.g., *Anolis oculatus*, Malhotra & Thorpe, 1997; *Phrynocephalus vlangalii*, Jin, Liu, & Li, 2007; *Sitana ponticeriana*, Puthal & Sahoo, 2014; *Morethia boulengeri*, Michael et al., 2014). Males had, on average, larger values than females for most morphological traits, with the morphological traits of males being between 0.08% (NarEye) and 28.7% (TailH) greater than those of females (Table S2). SVL in males was 2.0% greater than in females (Table 1). Three alternative nonmutually exclusive hypotheses, sexual selection favoring large size in males (owing to male–male competition or female choice), resource partitioning favoring small size in both sexes, and fecundity selection favoring large size in females (larger females produce more eggs), have been proposed to explain the evolution of sexual size dimorphism (Calderón‐Espinosa, Ortega‐León, & Zamora‐Abrego, 2013; Katsikaros & Shine, 1997; Stillwell, Blanckenhorn, Teder, Davidowitz, & Fox, 2010). In this study, we did not investigate the association between head size and diet, but Qiu et al. (2001) demonstrated that there is no significant food niche divergence between the two sexes in *C. versicolor*, suggesting that intraspecific resource partitioning is unlikely to be an alternative driving force. Adult males with a larger head size could have an advantage in intrasexual competition for territory defense and mate choice in this and other lizard species (Radder et al., 2001). Thus, according to these predictions, male competition (i.e., sexual selection) is a plausible for driver of sexual differences observed in body size variation. However, further studies are required to test this assumption.

### 4.2 Converse Bergmann's rule

The present results demonstrated that *C. versicolor* failed to fit Bergmann's rule, showing decreases or no significant change in body size variation with increasing latitude. The patterns found for females in this study are in agreement with those previously reported in ectotherms showing converse Bergmann clines (e.g., Boaratti & Da Silva, 2016; Jin et al., 2007; Mousseau, 1997; Muñoz et al., 2014). This is unsurprising because Bergmann's rule in ectotherms has been widely controversial (Blackburn et al., 1999; Boaratti & Da Silva, 2016; Olalla‐Tárraga, 2011). The “converse Bergmann's rule” predicted that smaller body sizes facilitate higher heating rates in colder environments and a positive association between temperature and size (Aragón & Fitze, 2014). Body temperature is highly influenced by the ambient temperature in an ectotherm. Individuals with a smaller body size warm and cool more rapidly due to their greater surface area‐to‐volume ratio. Thus, they can thermoregulate their body temperatures more precisely in cold environments (Muñoz et al., 2014). Individuals with a small body size may be adapted for thermoregulation at high latitude (Muñoz et al., 2014). These thermoregulatory physiological processes might not fit the original hypothesis of heat conservation. Furthermore, the ability of Bergmann's rule to explain intraspecific gradients in body size variation is still debated. The origin of the rule was a comparison of different (but closely related) species, although Bergmann himself later stated (Mayr, 1963; Rensch, 1938) that Bergmann's rule is also met at an intraspecific level (Boaratti & Da Silva, 2016; Vinarski, 2014). Some authors suggested that the intraspecific and interspecific body variations are rather distinct phenomena and should be explained by different mechanisms (Boaratti & Da Silva, 2016; Vinarski, 2014). In line with this view, we found that the body size variation in *C. versicolor* females showed a converse Bergmann clines, while the variation in *C. versicolor* males displayed no cline.

### 4.3 Correlations with body size variation

Climate change is predicted to have a large impact on the distributions of fauna (Hileman et al., 2017). Reptiles may be particularly vulnerable due to their thermal constraints and life cycles (Hileman et al., 2017). Our results suggest that multiple factors might affect geographic variation of body size in *C. versicolor*. Variation in the body size of *C. versicolor* males and females showed different relationships with each environmental variable, but in both involved mechanisms related to the heat balance and water availability hypotheses. The divergent predictors of patterns of body size variation between males and females suggest that different evolutionary selection pressures may be acting on them. Although it was assumed that correlations with body size variation patterns at both levels were similar, these comparisons should be interpreted with caution.

Ectotherms have certain physiological or behavioral mechanisms for regulating body temperature (Lindsey, 1966). Unfavorable climates would have dramatic consequences for ecological performance (imposed by natural selection) and reproductive success (imposed by sexual selection) in lizards because most physiological functions (and hence behavioral responses) would occur under such conditions at a suboptimal metabolic rate (Pianka & Vitt, 2003; Pincheira‐Donoso et al., 2008). Our results suggest that climate seasonality is negatively correlated with body size in males. Temperature seasonality reflects the degree of temperature fluctuation within a year (the larger the temperature range in this area is, the stronger the degree of climate fluctuation will be). In addition, climate seasonality dictates periodic departures from the climatic optima of organisms and thus favors species with more flexible adaptations to unevenly distributed resources or conditions (Gouveia et al., 2013; Klopfer, 1959; Stevens, 1989). The evolutionary cost of these adaptations would lead to seasonality playing a role in a progressive environmental filter with increases in climate variability increases (Gouveia et al., 2013). Potential effects of seasonality may reflect longer periods for growing due to extended activity (Aragón & Fitze, 2014; Mousseau, 1997). Although lizards may adjust (plastically or genetically) some physiological and behavioral traits to reduce stress and increase heating rates in dramatically changing environments, the efficiency of these adaptations is limited (Pincheira‐Donoso et al., 2008). Furthermore, fewer lizards would be able to overcome more variable climate conditions because tropical species have evolved under a narrower climatic regime (Gouveia et al., 2013). Thus, this slower growth at higher temperature ranges may produce a smaller adult body size as the lizards seek to consume more energy to maintain their body temperature and reduce maturity or fecundity in order to cope with dramatic temperature changes. This is in line with the findings of previous studies conducted in lizards (Ashton & Feldman, 2003; Horváthová et al., 2013; Pincheira‐Donoso et al., 2008).

Most studies have suggested that variables related to temperature would play a more important role in explaining reptile body size variation, while the effect of water‐related factors is more complex (Volynchik, 2014). However, body size variation in females showed a positive association with precipitation in the present study. This result have also found in some squamates (e.g., *Morethia boulengeri*, Michael et al., 2014; *Sistrurus catenatus*, Hileman et al., 2017). Different precipitation needs in a specific quarter may be due to restrictive ecological requirements, indicating that females of *C. versicolor* adapted to different amounts of precipitation during different growth periods, especially during the reproductive period and incubation of eggs. Heat loss is much more rapid in wetter habitats; thus, small species may not be able to maintain sufficient heat to survive under these situations (Meiri, 2008). By contrast, precipitation directly correlates with the primary production of plant communities and thus may influence the availability and abundance of prey (Hileman et al., 2017; Huston & Wolverton, 2011; Madsen & Shine, 2000). Prey availability and abundance during the growing season may substantially affect organism ontogenesis and survival, and are among the possible determinants of body size in reptiles (Berrigan & Charnov, 1994; Kaliontzopoulou, Adams, van der Meijden, Perera, & Carretero, 2012; Krause, Burghardt, & Gillingham, 2003). Better food resources are generally available for lizards at moister sites at the population level; thus, in nutrition levels, this may result in morphometric differentiation between individuals inhabiting dry or wet sites (Volynchik, 2014). However, further detailed investigation is required to examine whether prey variation between populations is enough to impact adult body size.

## 5 CONCLUSION

Our study is one of few to investigate intraspecific variation in *C. versicolor* life‐cycle traits over the geographic range of this species. We found significant variation in body size among populations of *C. versicolor*. Sexual size dimorphism is a widespread phenomenon and was evident in *C. versicolor* adults. Most body parts in males were larger than those in females. Male competition might be a plausible for driver of the sexual differences. Furthermore, geographic variation in body size in *C. versicolor* was consistent with the converse Bergmann's rule in females and showed no cline in males. However, the variation in body size in both sexes correlated with different environmental factors. Larger adult body size in females most likely occurred where there is more precipitation during the warmest quarter, while males in localities with lower seasonal temperature were larger than those in areas with higher seasonal temperature. We speculate that these differences may reflect an ecologically selected adaptation. However, this should be cautioned that, in comparison with the overall species range of *C. versicolor*, our sample sizes might be small. Thus in future studies, we recommend a combination of sampling more populations and more individuals at each site across the species range and more comprehensive hypotheses to interpret body size variations in *C. versicolor*.

## CONFLICT OF INTEREST

None declared.

## AUTHOR CONTRIBUTIONS

Conceived and designed the experiments: Yong Huang and Xiaomei Wei. Performed the experiments: Yong Huang and Xiaomei Wei. Analyzed the data: Yong Huang and Xiaomei Wei. Contributed reagents/materials/analysis tools: Linmiao Yan, Chengjian Zhao, Yueyun Zhang, Yongli Xu, and Ni Jiang. Wrote the manuscript: Yong Huang and Xiaomei Wei.
