# Supplementary material for: Geographic variation in body size and its relationship with environmental gradients in the Oriental Garden Lizard, Calotes versicolor
Source: Ecol Evol. 2018 Apr 2;8(9):4443–54. doi: 10.1002/ece3.4007 (PMC5938448; doi:10.1002/ece3.4007)
Supplement: Supplementary file 1 [file ECE3-8-4443-s001.doc]

Table S1 Loading of the first three principal components among 23 morphological traits of males (N=91) and females (N=89) of *Calotes versicolor*.

| Character | Males | | |  | Females | | |
| --- | --- | --- | --- | --- | --- | --- | --- |
|  | PC1 | PC2 | PC3 |  | PC1 | PC2 | PC3 |
| SVL | 0.94 | -0.17 | -0.07 |  | 0.82 | 0.37 | 0.03 |
| HeadL | 0.88 | 0.02 | -0.09 |  | 0.80 | -0.03 | -0.01 |
| HeadH | 0.85 | -0.15 | 0.07 |  | 0.77 | 0.17 | -0.40 |
| HeadW | 0.94 | -0.16 | 0.00 |  | 0.84 | 0.27 | -0.28 |
| JawW | 0.87 | -0.32 | -0.14 |  | 0.65 | 0.51 | -0.13 |
| SnW | 0.82 | 0.11 | -0.15 |  | 0.71 | -0.15 | -0.23 |
| Interorb | 0.62 | 0.58 | -0.25 |  | 0.67 | -0.55 | 0.17 |
| EyeL | 0.38 | -0.81 | -0.12 |  | 0.19 | 0.91 | -0.04 |
| SnEye | 0.73 | 0.57 | 0.06 |  | 0.68 | -0.63 | 0.05 |
| NarEye | 0.47 | 0.76 | 0.09 |  | 0.55 | -0.75 | 0.03 |
| EyeEar | 0.81 | 0.36 | -0.07 |  | 0.71 | -0.50 | -0.13 |
| SnForel | 0.84 | -0.13 | -0.09 |  | 0.63 | 0.42 | -0.07 |
| TailL | 0.38 | -0.08 | 0.55 |  | 0.62 | 0.15 | 0.16 |
| TailH | 0.72 | -0.05 | -0.48 |  | 0.72 | 0.03 | -0.41 |
| TailW | 0.57 | -0.05 | -0.40 |  | 0.77 | -0.05 | -0.40 |
| ForefL | 0.90 | -0.21 | 0.05 |  | 0.65 | 0.44 | 0.38 |
| 4FingLng | 0.57 | -0.41 | 0.25 |  | 0.44 | 0.34 | 0.43 |
| UpArmL | 0.79 | 0.20 | 0.01 |  | 0.67 | -0.20 | 0.46 |
| LoArmL | 0.90 | 0.01 | 0.02 |  | 0.80 | -0.15 | 0.08 |
| HindfL | 0.89 | -0.24 | 0.15 |  | 0.83 | 0.20 | 0.28 |
| 4ToeLn | 0.62 | 0.05 | 0.09 |  | 0.25 | 0.18 | 0.27 |
| UpLegL | 0.75 | 0.12 | 0.38 |  | 0.73 | -0.22 | 0.12 |
| CrusL | 0.67 | 0.05 | 0.37 |  | 0.77 | -0.05 | 0.09 |
| Cumulative%variance explained | 56.80 | 68.00 | 73.32 |  | 46.81 | 62.29 | 68.52 |

Character：SVL,snout-vent length; HeadL, head length; HeadH, head height; HeadW, head width; JawW, jaw width; SnW, snout width; Interorb, interorbital width; EyeL,eye length; SnEye, snout-eye length; NarEye, naris-eye length; EyeEar,eye-ear length; SnForel, snout-forelimb length; TailL, tail length; TailH, tail height; TailW, tail width; ForefL, forefoot length; 4FingLng, 4th finger; UpArmL, upper arm length; LoArmL, lower arm length; HindfL, hindfoot length; 4ToeLn, 4th toe; UpLegL, upper leg length; CrusL, crus length. PC1, PC2 and PC3, first three components by principal component analysis based on a correlation matrix of the data considering all above characters.

Table S2 Morphological data: Mean ± S.E.M. of male (N=91) and female (N=89) adults in 40 populations of *Calotes versicolor*.

| Number | Populations | Latitude | longitude | N | SVL | HeadL | HeadH | HeadW | JawW | SnW | Interorb | EyeL | SnEye | NarEye | EyeEar | SnForel | TailL | TailH | TailW | ForefL | 4FingLng | UpArmL | LoArmL | HindfL | 4ToeLn | UpLegL | CrusL |
| --- | --- | --- | --- | --- | --- | --- | --- | --- | --- | --- | --- | --- | --- | --- | --- | --- | --- | --- | --- | --- | --- | --- | --- | --- | --- | --- | --- |
| 1 | Tianya | 18.31 | 109.27 | 4 | 85.49±1.19 | 22.98±0.61 | 12.73±0.21 | 13.98±0.27 | 12.98±0.12 | 5.09±0.19 | 9.30±0.62 | 5.86±0.28 | 9.06±0.7 | 5.36±0.33 | 5.61±0.29 | 28.33±0.97 | 253.92±4.91 | 8.44±0.41 | 7.99±0.48 | 40.82±1.12 | 11.47±0.67 | 15.46±0.45 | 13.16±0.40 | 63.47±1.66 | 17.69±0.53 | 20.02±0.46 | 18.04±0.46 |
|  |  |  |  | 4 | 90.97±4.13 | 24.90±1.14 | 15.10±0.93 | 15.50±0.87 | 14.37±0.71 | 5.62±0.33 | 9.55±0.75 | 6.47±0.63 | 8.73±0.73 | 5.44±0.7 | 6.55±0.58 | 30.03±0.7 | 235.85±25.47 | 11.06±0.62 | 9.73±0.65 | 43.93±1.56 | 11.40±0.68 | 15.55±1.26 | 14.50±0.52 | 67.20±2.20 | 20.76±2.07 | 20.93±0.83 | 20.11±0.55 |
| 2 | Zhizhong | 18.63 | 109.29 | 3 | 80.71±0.63 | 20.94±0.68 | 11.98±0.35 | 13.8±0.39 | 12.64±0.36 | 5.14±0.24 | 9.06±0.86 | 5.57±0.63 | 8.31±0.34 | 4.92±0.31 | 6.12±0.39 | 28.04±0.64 | 245.59±8.78 | 7.99±0.47 | 8.12±0.42 | 37.42±1.10 | 10.68±0.19 | 13.86±0.86 | 13.27±0.43 | 59.88±0.36 | 16.06±0.28 | 19.42±0.34 | 17.11±0.48 |
|  |  |  |  | 1 | - | - | - | - | - | - | - | - | - | - | - | - | - | - | - | - | - | - | - | - | - | - | - |
| 3 | Jianfeng | 18.70 | 108.81 | 2 | 88.03±0.87 | 23.06±0.30 | 12.42±0.08 | 14.62±0.33 | 13.65±0.35 | 5.29±0.02 | 8.39±0.17 | 6.67±0.09 | 8.56±0.12 | 4.75±0.21 | 5.30±0.45 | 30.75±1.25 | 264.33±* | 7.99±0.39 | 7.71±0.62 | 40.97±0.58 | 11.38±0.13 | 15.64±0.22 | 13.32±0.22 | 63.44±0.75 | 16.00±0.20 | 20.31±0.11 | 18.29±0.31 |
|  |  |  |  | 2 | 77.12±1.50 | 21.73±0.13 | 11.88±0.01 | 13.06±0.20 | 12.07±0.23 | 4.75±0.16 | 8.83±0.78 | 4.72±0.20 | 8.92±0.68 | 5.82±0.57 | 5.86±0.34 | 28.70±1.29 | 234.17±3.72 | 9.36±0.73 | 8.47±0.07 | 37.53±0.41 | 10.42±0.1 | 13.65±0.35 | 11.63±0.43 | 59.72±0.69 | 16.85±0.01 | 18.44±0.41 | 16.80±0.50 |
| 4 | Baoyou | 18.76 | 109.08 | 6 | 76.31±1.68 | 20.38±0.36 | 11.63±0.29 | 12.84±0.24 | 11.91±0.26 | 4.72±0.13 | 7.94±0.83 | 5.24±0.36 | 8.20±0.39 | 4.88±0.33 | 4.98±0.18 | 25.21±0.63 | 232.09±4.52 | 7.00±0.25 | 7.13±0.38 | 36.70±1.35 | 9.90±0.27 | 14.29±0.62 | 12.52±0.42 | 57.57±1.47 | 15.81±0.32 | 18.95±0.73 | 16.35±0.32 |
|  |  |  |  | 2 | 78.89±0.57 | 16.31±4.38 | 13.03±0.61 | 13.56±0.30 | 12.77±0.17 | 4.80±0.04 | 7.79±0.15 | 6.20±0.55 | 7.70±0.25 | 4.78±0.04 | 5.06±0.28 | 28.43±1.35 | 238.51±* | 9.09±0.71 | 8.42±0.39 | 38.07±0.46 | 10.80±0.40 | 13.01±0.39 | 12.81±0.42 | 61.77±0.42 | 16.30±0.10 | 19.28±0.10 | 17.97±0.33 |
| 5 | Jianbian | 18.82 | 109.06 | 1 | - | - | - | - | - | - | - | - | - | - | - | - | - | - | - | - | - | - | - | - | - | - | - |
| 6 | Hongshan | 18.86 | 109.53 | 2 | 81.93±2.63 | 21.90±0.13 | 13.21±0.01 | 14.75±0.56 | 14.98±0.46 | 5.16±0.22 | 8.13±0.07 | 6.87±0.43 | 7.58±0.02 | 4.15±0.25 | 5.34±0.42 | 26.75±1.19 | 273.31±1.31 | 7.69±0.04 | 7.14±0.01 | 41.40±0.95 | 12.29±0.38 | 13.80±0.72 | 12.91±0.63 | 66.55±0.63 | 19.00±0.22 | 19.67±0.53 | 19.12±0.19 |
|  |  |  |  | 3 | 88.21±3.51 | 24.73±0.99 | 14.33±0.75 | 16.55±1.33 | 15.71±0.54 | 5.72±0.23 | 6.91±0.66 | 6.95±0.26 | 8.59±0.5 | 4.95±0.25 | 5.97±0.3 | 30.11±1.11 | 287.89±* | 8.99±0.42 | 9.24±0.28 | 44.34±0.81 | 13.46±1.46 | 15.05±0.39 | 14.41±0.27 | 68.98±1.25 | 19.60±0.28 | 21.83±0.75 | 20.28±0.32 |
| 7 | Fanyang | 18.88 | 109.36 | 5 | 86.44±1.33 | 23.48±0.55 | 12.77±0.52 | 14.25±0.5 | 14.07±0.33 | 5.47±0.08 | 9.96±0.72 | 5.91±0.48 | 9.30±0.39 | 5.75±0.55 | 5.98±0.25 | 28.22±1.15 | 235.86±22.79 | 7.53±0.22 | 7.61±0.42 | 40.64±0.62 | 13.29±1.24 | 15.54±0.63 | 13.38±0.21 | 63.56±1.34 | 16.87±1.02 | 20.46±0.57 | 18.65±0.51 |
| 8 | Wangxiaxiang | 19.01 | 109.14 | 8 | 82.55±1.48 | 22.57±0.55 | 11.68±0.28 | 13.72±0.26 | 13.04±0.4 | 5.21±0.07 | 9.63±0.6 | 5.36±0.25 | 9.21±0.54 | 5.49±0.38 | 5.73±0.39 | 25.60±0.74 | 241.91±3.99 | 7.54±0.44 | 7.83±0.32 | 39.90±1.14 | 10.88±0.23 | 15.06±0.64 | 13.50±0.34 | 62.64±0.68 | 17.07±0.22 | 19.59±0.36 | 18.26±0.37 |
| 9 | Donghe | 19.02 | 108.99 | 4 | 84.89±0.64 | 24.05±0.23 | 13.08±0.06 | 14.23±0.26 | 13.67±0.22 | 5.31±0.11 | 10.25±0.07 | 5.91±0.12 | 8.94±0.14 | 5.48±0.13 | 6.18±0.19 | 30.41±0.69 | 262.35±4.49 | 10.21±0.29 | 9.27±0.35 | 40.5-±0.85 | 10.97±0.45 | 15.28±0.46 | 13.41±0.32 | 62.84±0.62 | 17.59±0.31 | 20.79±0.58 | 18.64±0.21 |
| 10 | Bawangling | 19.03 | 109.12 | 3 | 84.35±0.42 | 23.26±0.61 | 12.17±0.22 | 13.80±0.23 | 13.13±0.24 | 5.42±0.18 | 9.22±0.71 | 6.15±0.59 | 9.07±0.54 | 5.37±0.5 | 6.14±0.45 | 29.17±1.28 | 245.71±7.63 | 8.21±0.65 | 8.13±0.17 | 41.53±0.67 | 12.23±0.45 | 15.45±0.45 | 13.53±0.16 | 64.13±0.80 | 17.07±0.5 | 18.92±0.35 | 18.53±0.40 |
|  |  |  |  | 4 | 78.94±0.48 | 22.70±1.20 | 12.19±0.25 | 13.90±0.18 | 13.25±0.29 | 5.11±0.20 | 9.11±1.11 | 5.52±0.44 | 8.49±0.46 | 5.18±0.58 | 5.49±0.7 | 28.41±0.11 | 236.11±7.73 | 8.64±0.26 | 8.39±0.22 | 39.99±0.75 | 10.97±0.30 | 14.43±0.25 | 13.00±0.52 | 61.46±1.36 | 17.84±0.12 | 19.33±0.39 | 18.32±0.14 |
| 11 | Hongmao | 19.03 | 109.67 | 4 | 84.59±2.73 | 22.84±0.8 | 12.89±0.35 | 14.59±0.43 | 13.66±0.54 | 5.45±0.15 | 8.29±0.25 | 6.77±0.18 | 7.95±0.19 | 4.82±0.12 | 5.39±0.07 | 27.53±0.93 | 134.88±4.90 | 8.51±0.22 | 134.71±8.38 | 265.36±5.16 | 40.26±0.75 | 11.87±0.19 | 16.80±0.14 | 8.77±0.45 | 62.59±1.18 | 13.82±0.46 | 13.60±0.41 |
|  |  |  |  | 4 | 82.33±3.71 | 23.08±1.00 | 13.40±0.51 | 15.51±0.63 | 14.87±0.77 | 5.50±0.13 | 8.96±0.35 | 6.74±0.25 | 8.23±0.41 | 5.14±0.20 | 5.83±0.25 | 29.27±1.24 | 272.1±7.65 | 9.19±0.14 | 8.48±0.41 | 42.39±1.70 | 12.08±0.51 | 14.12±0.88 | 13.88±0.40 | 66.33±1.78 | 20.22±1.210 | 21.47±1.31 | 20.89±0.87 |
| 12 | Datian | 19.12 | 108.83 | 5 | 86.08±0.97 | 20.99±1.55 | 13.32±0.3 | 14.90±0.36 | 13.84±0.54 | 5.38±0.07 | 9.08±0.63 | 6.31±0.53 | 8.95±0.5 | 5.34±0.42 | 6.40±0.28 | 27.77±0.71 | 231.66±4.05 | 7.93±0.17 | 8.18±0.28 | 39.31±0.67 | 10.63±0.11 | 13.83±0.48 | 13.66±0.14 | 61.23±1.07 | 16.64±0.37 | 19.09±0.82 | 17.92±0.34 |
|  |  |  |  | 1 | - | - | - | - | - | - | - | - | - | - | - | - | - | - | - | - | - | - | - | - | - | - | - |
| 13 | Qiongzhong | 19.13 | 109.91 | 3 | 75.48±5.35 | 20.98±1.43 | 12.5±0.84 | 13.56±1.03 | 12.80±1.33 | 4.99±0.27 | 7.81±0.3 | 6.78±0.37 | 7.46±0.16 | 4.13±0.15 | 5.00±0.54 | 25.66±2.1 | 259.17±14.27 | 8.24±0.56 | 8.53±0.67 | 40.03±2.00 | 12.09±0.62 | 14.34±1.01 | 12.20±0.57 | 63.08±3.56 | 18.51±1.25 | 19.11±1.6 | 18.36±0.80 |
| 14 | Huangzhu | 19.44 | 19.44 | 1 | - | - | - | - | - | - | - | - | - | - | - | - | - | - | - | - | - | - | - | - | - | - | - |
| 15 | Tunchang | 19.58 | 110.18 | 1 | - | - | - | - | - | - | - | - | - | - | - | - | - | - | - | - | - | - | - | - | - | - | - |
| 16 | Fushan | 19.87 | 109.92 | 1 | - | - | - | - | - | - | - | - | - | - | - | - | - | - | - | - | - | - | - | - | - | - | - |
| 17 | Haikou | 20.00 | 110.34 | 2 | 85.09±5.09 | 22.41±1.53 | 13.65±0.23 | 14.29±0.63 | 13.52±0.64 | 5.50±0.05 | 8.34±0.37 | 7.16±0.73 | 8.36±0.4 | 4.67±0.22 | 5.40±0.02 | 29.65±2.11 | 247.71±* | 7.47±0.71 | 8.20±0.03 | 40.62±0.27 | 12.33±0.07 | 14.27±0.27 | 12.53±0.22 | 62.64±0.36 | 16.19±0.71 | 19.96±0.23 | 18.48±0.47 |
| 18 | Weizhoudao | 21.06 | 109.11 | 6 | 90.16±2.94 | 23.62±0.56 | 13.40±0.49 | 15.89±0.28 | 15.72±0.45 | 5.42±0.2 | 9.25±0.37 | 7.32±0.22 | 8.16±0.23 | 4.89±0.27 | 5.90±0.2 | 30.48±0.78 | 259.74±3.04 | 9.76±0.57 | 9.11±0.55 | 41.15±0.56 | 12.02±0.31 | 15.23±0.34 | 13.55±0.32 | 64.06±1.2- | 17.31±0.49 | 19.95±0.22 | 18.51±0.32 |
|  |  |  |  | 13 | 93.11±1.28 | 25.58±0.43 | 14.25±0.23 | 17.01±0.31 | 16.54±0.42 | 6.09±0.14 | 9.81±0.19 | 7.41±0.17 | 8.68±0.12 | 4.72±0.09 | 6.41±0.17 | 31.81±0.49 | 243.83±13.38 | 11.55±0.46 | 9.86±0.29 | 44.68±0.55 | 13.16±0.44 | 15.73±0.27 | 14.58±0.23 | 67.92±0.72 | 18.12±0.49 | 19.92±0.92 | 19.88±0.35 |
| 19 | Yinhai | 21.47 | 109.08 | 2 | 91.40±0.81 | 23.83±0.02 | 13.71±0.25 | 16.72±0.94 | 16.93±1.2 | 5.55±0.3 | 9.75±0.21 | 7.58±0.60 | 8.55±0.3 | 4.82±0.16 | 5.71±0.13 | 30.51±1.95 | 245.64±* | 9.70±0.20 | 8.07±0.27 | 42.79±0.24 | 13.46±0.32 | 15.84±0.31 | 14.20±0.09 | 65.23±1.56 | 17.29±0.21 | 21.35±0.72 | 18.50±0.38 |
|  |  |  |  | 1 | - | - | - | - | - | - | - | - | - | - | - | - | - | - | - | - | - | - | - | - | - | - | - |
| 20 | Gangkou | 21.64 | 108.30 | 2 | 84.09±5.23 | 21.42±0.94 | 12.14±0.71 | 14.63±0.71 | 15.68±0.94 | 4.73±0.15 | 8.63±0.49 | 6.95±0.45 | 7.98±0.39 | 4.19±0.17 | 4.72±0.36 | 25.53±1.48 | 238.36±5.30 | 7.72±0.22 | 8.09±0.40 | 41.83±1.12 | 13.04±0.69 | 14.61±0.21 | 12.10±0.45 | 62.78±0.07 | 17.46±0.3 | 19.09±0.19 | 17.80±0.82 |
|  |  |  |  | 1 | - | - | - | - | - | - | - | - | - | - | - | - | - | - | - | - | - | - | - | - | - | - | - |
| 21 | Shiwandashan | 21.91 | 107.92 | 1 | - | - | - | - | - | - | - | - | - | - | - | - | - | - | - | - | - | - | - | - | - | - | - |
| 22 | Qinnan | 21.98 | 108.65 | 5 | 90.41±3.67 | 24.16±0.58 | 13.2±0.54 | 15.48±0.63 | 16.13±0.55 | 5.27±0.22 | 8.64±0.29 | 7.08±0.20 | 8.48±0.17 | 4.37±0.12 | 5.51±0.27 | 28.84±0.82 | 252.09±6.05 | 9.89±0.72 | 8.92±0.55 | 42.96±0.96 | 12.52±0.48 | 16.17±0.36 | 13.29±0.49 | 63.81±0.87 | 17.92±0.19 | 19.74±0.51 | 18.64±0.43 |
|  |  |  |  | 5 | 92.79±3.55 | 24.61±0.96 | 13.37±0.43 | 16.13±0.63 | 16.58±.67 | 5.55±0.17 | 8.86±0.32 | 7.46±0.21 | 8.37±0.18 | 4.54±0.14 | 5.64±0.29 | 31.95±1.77 | 254.12±6.36 | 10.56±0.56 | 8.77±0.29 | 46.08±1.85 | 13.72±0.44 | 16.43±0.68 | 14.00±0.86 | 68.52±2.22 | 19.69±0.58 | 20.89±1.01 | 19.81±0.53 |
| 23 | Buguan | 22.07 | 106.79 | 1 | - | - | - | - | - | - | - | - | - | - | - | - | - | - | - | - | - | - | - | - | - | - | - |
| 24 | Rongxi | 22.78 | 110.44 | 2 | 72.44±1.42 | 19.34±0.34 | 10.26±0.1 | 11.70±0.07 | 12.30±0.11 | 4.57±0.26 | 7.40±0.03 | 5.98±0.13 | 6.37±0.15 | 3.28±0.02 | 4.08±0.05 | 24.54±0.46 | 205.87±0.56 | 6.11±0.02 | 6.02±0.39 | 36.66±0.64 | 11.38±0.39 | 12.43±0.15 | 10.93±0.66 | 55.94±1.10 | 15.90±0.26 | 16.19±0.23 | 15.46±0.11 |
|  |  |  |  | 3 | 92.31±7.52 | 23.45±1.53 | 13.35±1.22 | 15.30±11.5 | 15.69±1.3 | 5.67±0.42 | 9.12±0.76 | 7.71±0.19 | 8.32±0.56 | 4.24±0.37 | 6.15±0.59 | 32.20±2.49 | 250.28±15.37 | 10.44±0.76 | 9.77±1.07 | 45.27±1.87 | 13.01±0.78 | 15.28±0.77 | 13.65±0.59 | 66.60±3.17 | 18.76±0.83 | 20.09±1.56 | 18.00±1.28 |
| 25 | Cenxi | 22.91 | 110.96 | 1 | - | - | - | - | - | - | - | - | - | - | - | - | - | - | - | - | - | - | - | - | - | - | - |
|  |  |  |  | 3 | 84.71±6.42 | 21.07±1.65 | 12.51±0.7 | 14.34±1.17 | 14.46±1.54 | 4.98±0.44 | 8.53±0.48 | 7.78±0.54 | 7.93±0.64 | 3.80±0.43 | 5.13±0.64 | 30.54±1.77 | 244.19±13.07 | 10.58±0.83 | 7.76±0.69 | 41.99±2.43 | 12.23±0.42 | 14.77±0.8 | 12.83±0.95 | 61.77±3.04 | 18.47±0.69 | 19.56±1.05 | 17.16±0.35 |
| 26 | Wutang | 22.95 | 108.56 | 2 | 78.03±5.18 | 20.20±0.81 | 11.73±0.91 | 13.45±0.41 | 13.71±0.66 | 5.25±0.27 | 8.32±0.71 | 7.34±0.51 | 7.18±0.2 | 4.10±0.02 | 4.89±0.42 | 26.07±1.76 | 228.08±6.98 | 6.85±0.35 | 6.96±0.74 | 41.79±0.66 | 12.07±0.17 | 14.50±0.46 | 12.28±0.17 | 60.49±0.83 | 12.62±3.93 | 17.77±0.92 | 17.14±0.91 |
|  |  |  |  | 1 | - | - | - | - | - | - | - | - | - | - | - | - | - | - | - | - | - | - | - | - | - | - | - |
| 27 | Tiandeng | 23.09 | 107.15 | 3 | 78.73±5.76 | 20.69±0.8 | 10.2±0.79 | 13.84±0.53 | 14.14±0.28 | 4.84±0.03 | 7.45±0.35 | 6.30±0.40 | 7.06±0.1 | 3.76±0.13 | 4.84±0.19 | 27.39±1.57 | 198.44±11.02 | 7.35±0.53 | 6.94±0.25 | 38.13±1.66 | 11.49±0.56 | 13.48±0.67 | 11.86±0.22 | 58.87±2.18 | 17.23±0.64 | 17.26±1.22 | 16.88±0.42 |
|  |  |  |  | 1 | - | - | - | - | - | - | - | - | - | - | - | - | - | - | - | - | - | - | - | - | - | - | - |
| 28 | Gangbei | 23.09 | 109.54 | 1 | - | - | - | - | - | - | - | - | - | - | - | - | - | - | - | - | - | - | - | - | - | - | - |
|  |  |  |  | 4 | 80.10±0.64 | 20.59±0.23 | 11.94±0.06 | 13.71±0.26 | 13.62±0.22 | 5.02±0.11 | 8.33±0.07 | 6.73±0.12 | 7.53±0.14 | 4.00±0.13 | 4.44±0.19 | 26.38±0.69 | 228.20±4.49 | 10.80±0.29 | 9.02±0.35 | 40.51±0.85 | 11.46±0.45 | 13.70±0.46 | 12.27±0.32 | 61.43±0.62 | 17.55±0.31 | 18.62±0.58 | 17.75±0.21 |
| 29 | Dingdang | 23.13 | 107.98 | 3 | 82.84±4.03 | 20.88±0.25 | 10.75±0.21 | 12.98±0.42 | 14.31±0.58 | 5.19±0.18 | 8.14±0.08 | 7.19±0.31 | 7.56±0.27 | 4.33±0.17 | 4.84±0.21 | 28.09±1.26 | 234.68±9.32 | 6.72±0.38 | 6.44±0.45 | 39.42±0.56 | 12.48±0.61 | 11.89±0.11 | 12.06±0.53 | 58.51±1.09 | 17.19±0.82 | 16.47±0.35 | 16.73±0.57 |
|  |  |  |  | 3 | 83.79±5.00 | 20.18±1.25 | 11.15±0.66 | 13.16±0.79 | 13.09±0.82 | 5.13±0.29 | 8.70±0.66 | 7.17±0.07 | 6.96±0.72 | 4.22±0.03 | 5.41±0.73 | 26.48±1.69 | 235.34±15.04 | 7.97±0.20 | 7.20±0.35 | 39.68±2.68 | 11.26±0.60 | 13.40±0.78 | 11.44±0.85 | 59.64±2.23 | 17.53±0.41 | 16.72±0.75 | 17.22±0.67 |
| 30 | Yunchun | 23.52 | 108.53 | 3 | 84.01±6.03 | 22.80±1.88 | 12.61±0.75 | 14.99±1.03 | 15.1±0.99 | 4.81±0.22 | 8.78±0.54 | 6.98±0.4 | 7.80±0.41 | 4.11±0.24 | 5.74±0.53 | 30.69±2.99 | 267.6±7.47 | 9.24±0.71 | 9.61±0.69 | 42.98±2.83 | 13.14±0.89 | 15.05±0.79 | 13.72±1.12 | 65.25±4.35 | 17.68±0.84 | 19.67±2.22 | 18.52±1.5 |
| 31 | Damingshan | 23.53 | 108.34 | 1 | - | - | - | - | - | - | - | - | - | - | - | - | - | - | - | - | - | - | - | - | - | - | - |
|  |  |  |  | 5 | 74.56±1.97 | 20.49±0.49 | 11.19±0.48 | 12.85±0.29 | 13.74±0.38 | 4.75±0.07 | 7.63±0.25 | 6.37±0.11 | 6.89±0.19 | 3.94±0.17 | 4.79±0.19 | 29.39±1.61 | 215.96±4.55 | 9.52±0.31 | 8.53±0.17 | 37.30±0.76 | 10.93±0.3 | 12.65±0.31 | 11.21±0.29 | 57.98±1.27 | 16.26±0.36 | 16.88±0.48 | 15.96±0.42 |
| 32 | Wuzhou | 23.53 | 111.33 | 3 | 80.29±2.33 | 19.60±0.75 | 10.41±0.36 | 12.88±0.62 | 13.55±0.4 | 4.68±0.16 | 7.64±0.12 | 7.10±0.15 | 6.85±0.17 | 3.33±0.21 | 4.78±0.12 | 24.93±0.88 | 215.91±8.22 | 5.99±0.25 | 5.48±0.09 | 39.57±0.88 | 11.93±0.16 | 13.12±0.71 | 12.55±0.43 | 59.52±2.26 | 15.75±0.92 | 18.42±0.67 | 16.07±1.05 |
|  |  |  |  | 1 | - | - | - | - | - | - | - | - | - | - | - | - | - | - | - | - | - | - | - | - | - | - | - |
| 33 | Naheng | 23.95 | 107.07 | 1 | - | - | - | - | - | - | - | - | - | - | - | - | - | - | - | - | - | - | - | - | - | - | - |
| 34 | Bayan | 24.09 | 107.25 | 2 | 85.00±0.66 | 21.12±0.21 | 11.11±0.08 | 13.49±0.19 | 13.52±0.24 | 4.3±0.26 | 6.73±0.77 | 7.34±0.19 | 7.32±0.32 | 3.62±0.08 | 5.21±0.32 | 29.35±0.39 | 237.9±* | 6.57±0.60 | 7.51±0.30 | 41.15±0.54 | 13.13±0.27 | 13.95±0.57 | 12.33±0.16 | 60.42±0.05 | 17.74±0.2 | 17.22±0.17 | 16.42±0.36 |
|  |  |  |  | 3 | 84.80±5.32 | 22.29±1.42 | 12.20±0.78 | 14.03±0.77 | 15.36±1.5 | 5.25±0.46 | 8.55±0.67 | 7.58±0.37 | 7.77±0.39 | 4.09±0.4 | 5.35±0.28 | 29.06±1.5 | 224.5±16.77 | 9.98±0.66 | 9.66±0.89 | 41.81±2.76 | 12.01±0.32 | 13.71±1.31 | 12.78±0.45 | 63.04±3.31 | 18.12±0.63 | 18.03±1.30 | 14.00±2.15 |
| 35 | Lingyun | 24.21 | 106.61 | 3 | 95.45±1.47 | 22.95±1.19 | 12.06±0.74 | 14.74±0.89 | 15.57±0.84 | 5.25±0.33 | 9.08±0.51 | 8.04±0.18 | 7.79±0.44 | 4.06±0.32 | 5.39±0.43 | 31.85±0.26 | 250.11±* | 7.55±0.98 | 8.50±0.73 | 45.51±1.5 | 12.55±0.53 | 15.34±0.20 | 13.72±0.68 | 62.83±1.75 | 17.63±0.66 | 18.55±1.35 | 18.06±0.82 |
|  |  |  |  | 1 | - | - | - | - | - | - | - | - | - | - | - | - | - | - | - | - | - | - | - | - | - | - | - |
| 36 | Haian | 20.28 | 110.21 | 1 | - | - | - | - | - | - | - | - | - | - | - | - | - | - | - | - | - | - | - | - | - | - | - |
| 37 | Yangchun | 22.14 | 111.78 | 2 | 85.68±2.26 | 20.34±0.78 | 11.92±0.08 | 13.97±0.28 | 14.44±0.74 | 5.09±0.07 | 8.06±0.37 | 7.42±0.25 | 7.24±0.46 | 4.10±0.16 | 4.50±0.32 | 26.04±0.01 | 224.41±* | 6.59±0.34 | 7.09±0.33 | 40.48±0.77 | 12.49±0.43 | 13.73±0.44 | 11.62±0.19 | 61.24±0.21 | 16.95±0.09 | 18.12±0.34 | 17.17±0.22 |
|  |  |  |  | 3 | 88.6±1.81 | 24.22±1.26 | 13.65±0.73 | 14.68±0.65 | 15.58±0.86 | 5.47±0.06 | 9.35±0.31 | 7.67±0.18 | 8.03±0.33 | 4.18±0.34 | 5.67±0.33 | 31.34±0.96 | 275.73±12.23 | 10.05±0.43 | 12.29±2.43 | 41.44±0.94 | 11.72±0.57 | 13.61±0.55 | 14.43±0.29 | 64.83±1.63 | 18.38±0.53 | 19.36±0.88 | 18.92±0.86 |
| 38 | Xinyang | 22.34 | 110.94 | 2 | 87.54±2.49 | 21.37±0.72 | 12.10±0.25 | 13.5±0.15 | 14.00±0.59 | 4.93±0.12 | 9.19±0.41 | 7.66±0.34 | 7.90±0.23 | 4.59±0.03 | 5.11±0.28 | 30.28±1.84 | 233.30±12.71 | 7.66±0.62 | 7.17±0.05 | 41.29±2.49 | 12.25±0.48 | 13.86±1.27 | 13.07±0.08 | 60.62±2.14 | 17.45±0.31 | 17.43±0.63 | 17.28±0.01 |
|  |  |  |  | 3 | 88.89±4.78 | 23.37±1.18 | 12.80±0.81 | 14.20.98 | 14.51±0.95 | 5.27±0.23 | 8.95±0.48 | 8.07±0.46 | 7.83±0.46 | 3.94±0.28 | 5.56±0.41 | 31.74±2.6 | 230.44±24.1 | 10.12±0.70 | 8.21±0.65 | 43.65±2.76 | 12.32±0.47 | 14.77±1.13 | 14.08±0.68 | 65.83±2.79 | 18.77±0.81 | 19.19±1.49 | 18.64±0.80 |
| 39 | Shuikou | 22.43 | 106.64 | 1 | - | - | - | - | - | - | - | - | - | - | - | - | - | - | - | - | - | - | - | - | - | - | - |
| 40 | Luoding | 22.78 | 111.61 | 3 | 84.23±0.54 | 20.18±0.18 | 10.98±0.25 | 12.8±0.4 | 13.35±0.21 | 4.85±0.22 | 7.69±0.08 | 7.46±0.03 | 6.96±0.26 | 3.26±0.02 | 4.67±0.01 | 28.51±0.49 | 232.57±2.47 | 6.19±0.42 | 6.03±0.3 | 41.58±0.55 | 12.69±0.29 | 15.13±0.26 | 12.81±0.4 | 61.16±0.83 | 17.86±0.42 | 17.87±0.91 | 16.57±0.22 |
|  |  |  |  | 2 | 80.37±5.77 | 20.64±1.43 | 11.43±0.7 | 13.71.16 | 13.14±1.1 | 5.09±0.25 | 8.09±0.49 | 7.76±0.53 | 6.77±0.52 | 3.33±0.34 | 4.73±0.4 | 29.28±1.54 | 223.65±10.48 | 9.20±0.27 | 7.93±0.57 | 40.50±2.11 | 11.54±0.78 | 14.90±0.29 | 12.85±0.61 | 61.57±3.66 | 17.69±0.91 | 18.47±1.03 | 18.27±1.03 |

-, denotes data cannot be calculated due to one sample.

*, denotes S.E.M data cannot be calculated due to the tail was broken off.

SVL,snout-vent length; HeadL, head length; HeadH, head height; HeadW, head width; JawW, jaw width; SnW, snout width; Interorb, interorbital width; EyeL,eye length; SnEye, snout-eye length; NarEye, naris-eye length; EyeEar,eye-ear length; SnForel, snout-forelimb length; TailL, tail length; TailH, tail height; TailW, tail width; ForefL, forefoot length; 4FingLng, 4th finger; UpArmL, upper arm length; LoArmL, lower arm length; HindfL, hindfoot length; 4ToeLn, 4th toe; UpLegL, upper leg length; CrusL, crus length.
